# Improvement of Mechanical, Hydrophobicity and Thermal Properties of Chinese Fir Wood by Impregnation of Nano Silica Sol

**DOI:** 10.3390/polym12081632

**Published:** 2020-07-23

**Authors:** Enguang Xu, Yanjuan Zhang, Lanying Lin

**Affiliations:** 1 Research Institute of Wood Industry, Chinese Academy of Forestry, Beijing 100091, China; enguangxu@foxmail.com; 2 Jixian Honors College, Zhejiang Agriculture and Forestry University, Hangzhou 311300, China; fdlmy@163.com

**Keywords:** in-situ composition, nano silica sol, SiO_2_-wood composites, wood cell wall, vacuum/pressure impregnation

## Abstract

In this paper, a wood-SiO_2_ composite material was prepared via in-situ polymerization using vacuum/pressure impregnation technology using commercial scale nano silica sol and Chinese Fir (*Cunninghamia lanceolate* (Lamb.) Hook.). Scanning electron microscopy (SEM), Fourier transform infrared spectroscopy (FTIR), X-ray diffraction (XRD), thermogravimetric analysis (TG), and water contact angle were used to study the changes in the microstructure and physical and mechanical properties of this composite. The results showed that silica sol can penetrate and distribute into the wood cell cavities and surface of cell walls and hence combine with the substances of wood materials. FTIR results indicated that the –OH groups of wood can polycondense in-situ with silica sol to form Si–O–C covalent bonds, and amorphous SiO_2_ formed from Si–O–Si bonds between the –OH groups of silica sol did not change the crystalline structure of wood cell walls. This in-situ formulating composite significantly improved the compact microstructure, thermal and mechanical properties, and hydrophobicity of the composites.

## 1. Introduction

Wood is one of the oldest materials, mainly composed of cellulose, hemicellulose, and lignin, and is the only truly renewable material. Wood has excellent mechanical properties, unique decorative properties, and special environmental characteristics, widely used in architecture and interior decoration. However, some inherent inferior properties, e.g., lower strength and dimension changes over the exposing environments and easy to lose mechanical strength under attack by insects, have prevented efficient uses and exploitation of its full potential. To improve the strength and hardness, dimensional stability, and antiseptic properties, modifications with suitable chemicals have been employed and proven to be an attractive way [1,2]. In order to improve the physical and mechanical properties and weather resistance of wood, different scholars use various inorganic or organic modifiers to treat the wood [2,3,4].

However, the toxicity of modified products has caught more and more public attention. The development of nontoxic wood products has become vitally important [5]. Silicon is a kind of nontoxic and abundant element, which can exist in inorganic or organic compounds in various forms, and can be used for wood modification [6,7]. Petrified wood is a typical example, which shows that organic materials can be transformed into organic-inorganic composite materials under certain conditions [8]. This type of the SiO_2_-wood composite is one of the earliest wood modification methods. The double diffusion method is used to introduce the modifier into the wood to improve the wood performance, especially the wood’s fire resistance and decay resistances have been significantly improved [9,10,11,12]. Water glass (sodium silicate) is a common modifier that reacts with metalic salts to form inorganic deposits. If the sodium ions in water glass are replaced with aluminum sulfate (Al_2_ (SO_4_)_3_) and calcium chloride (CaCl_2_), the silicate component can be added to the interior of the wood by impregnation [9]. Other attempts for further improvement included barium chloride (BaCl_2_), boric acid (H_3_BO_3_), borax (Na_2_B_4_O_7_), boron trioxide (B_2_O_3_), and potassium borate (K_2_B_4_O_7_) [10,12]. These modified woods have high fungal resistance and fire resistance, but poor water resistance, because various salt components in water glass and the unreacted salt in the lumina of the wood cells have strong moisture absorption, and these modifiers have poor resistance to bleeding [10].

In order to improve the bleed resistance of inorganic salts, some scholars have begun to use the sol-gel method to prepare inorganic wood composite [6,7]. The main raw materials used in the sol-gel process are metal alkoxides, which are used as precursors, such as tetraethoxysilane (TEOS), 2-heptadecafluorooctylethyltrimet-hoxysilane (HFOETMOS), methyltrimethoxysilane (MTMOS), and polydimethylsiloxane (PDMS) [13,14,15,16,17,18,19]. Other reagents have also been investigated as precursors, including silicon alkoxides, silicon alkoxides derivatives, titanium alkoxides, and titanium alkoxides derivatives [20,21,22,23,24,25,26,27,28]. The precursor is dissolved in water or an organic solvent (methanol, ethanol, isopropanol, 2-methoxyethanol), and an acid or alkali is added as a hydrolysis catalyst so that the precursor can undergo hydrolysis reaction with the solvent or water in the wood; these hydrolysates are aggregated into nano-scale particles to produce a sol. The sol is introduced into the wood cell cavity, cell wall, and pit cavity by coating or vacuum pressure impregnation, then hydrolyzed and condensed at the same time. After aging, the sol becomes a gel with a three-dimensional network structure, and these gels are dried to form inorganic nano-oxides, which are deposited in the cell wall and cell cavity of the wood by hydrogen bonding or physical filling. The obtained modified wood has improved dimensional stability, fire resistance, and anti-leachability while retaining the porous structures characteristic of wood.

Nanosols (nano particulate inorganic sols) are another kind of wood modifier. It is a nano-scale transparent dispersion made by mixing inorganic particles with water or organic liquid. The diameter of the particles in the dispersion are less than 50 nm and the solid content is between 4% and 20% by weight [29]. Like other nanomaterials, it has a high surface-to-volume ratio, making it show greater activity in surface-related phenomena than bulky systems of the same mass. Common treatment methods include sol-gel, sonochemistry, solvothermal, nanopolymer carriers, combustion, chemical vapor deposition, and coating treatment. Among them, the sol-gel method is the most widely used and can introduce metal nanoparticles (gold, copper, and silver) and metal oxides (zinc, aluminum, and titania) into the wood [29]. However, the sol-gel method is difficult to popularize in industrial production, because the modifiers used are expensive, the moisture content of wood is strictly controlled, and the processing technology is complex.

Nano-silica sol is also one of these nanomaterials. There are many different ways to prepare nano-silica sol. The most commonly used methods are the ion exchange method, one-step hydrolysis of silica powder, and hydrolysis of silane. The ion exchange process is the most mature and widely used process. Nano-silica sol can be obtained by removing alkali in water glass using ion exchange technology. If the reaction continues, the nano-silica sol will eventually polymerize to form particles of amorphous silicon dioxide. Re-addition of alkali at an appropriate time stops the polycondensation reaction, so the unmodified nano-silica sol is usually alkaline. However, the nano aqueous silica sols used in the industry have undergone different surface modifications to become acidic or neutral [29,30]. Compared with other nanomaterials, these products are inexpensive and widely used in the fields of papermaking, ceramics, catalysts, abrasives, and solid-state electrochemical devices. In addition, silica sols are used in many coating applications to improve mechanical properties and anti-blocking, adhesion and wetting properties [31,32,33]. In addition, the high surface-to-weight ratio (≥200 m^2^/g) and nanoscale particle size of nano sols could be most compatible to the microstructural network system of wood; they can easily, effectively, and deeply penetrate into the wood substrates and a uniform distribution can be achieved.

In this study, a vacuum pressure impregnation method was used to introduce a commercial aqueous nano-silica sol into Chinese fir (*Cunninghamia lanceolate* (Lamb.) Hook). The applied nano-silica sol is uniformly filled in wood then nano particles are in situ synthesized on a wood surface by chemical reactions, to present a wood-SiO_2_ composite. The structure of the composite materials was observed, and the thermal and mechanical properties as well as the hydrophobicity of the composites were evaluated.

## 2. Materials and Methods

### 2.1. Materials

Fast-growing Chinese Fir (*Cunninghamia lanceolate* (Lamb.) Hook.) were collected from a plantation in Hangzhou, Zhejiang Province, China. Samples were taken from 0.3 m above the ground and then dried to 30% in a drying kiln. The dried timber was then cut into 300 × 70 × 70 mm blocks according to GB/T1928~1929-2009; all the samples were taken from the same tree to ensure the minimum difference in density and mechanical properties. Flat-sawn board was selected for the experiment and then conditioned in a climate chamber at 23 ± 2 °C and 65 ± 2% relative humidity (RH) until its equilibrium before silica sol impregnation.

Nano silica sol, an odorless, non-toxic colloid formed using SiO_2_ nanoparticles in water, whose molecular formula is mSiO_2_.nH_2_O, was purchased from Qingdao Hengshengda Chemical Co., Ltd., Qingdao, Shandong Province, China. Its pH value was 6.97, the solid content was 21.24%, the specific gravity was 1.153 g/cm^3^, the average size was 20.54 nm, and the dynamic viscosity was 1.87 × 10^−3^ Pa.s.

### 2.2. Preparation of SiO_2_-Wood Composites

According to the full cell impregnation process (Bethel process), a vacuum pressure impregnation tank is used to impregnate the wood. After the wood is placed in the cavity of the impregnation tank, a weight is used to press the wood to avoid the movement of the wood during the impregnation process. First, the cavity was evacuated and kept for 10 min, with a vacuum of −0.09 MPa. Then, the silica sol was added into the chamber and pressure impregnation was started. The pressure was 1.0 MPa and the holding time was 30, 60 and 90 min according to the experimental settings. After reaching the time, the silica sol was withdrawn from the cavity. Then, negative pressure (−0.09 MPa) was applied to the cavity for 10 min to remove unnecessary silica sol on the sample surface. After impregnation, all samples were dried at 103 °C for 24 h and then conditioned under 23 ± 2 °C and 65% relative humidity. The weight and dimension changes of the wood samples were recorded at each step of the experiment.

### 2.3. Characterization of SiO_2_-Wood Composites

#### 2.3.1. Weight Percent Gain (WPG)

The mass change of each sample was calculated according to the following formulas with all measurements taken at oven-dry state:

WPG (%) = (W_t_ − W_0_)/W_0_ × 100% (1)

where W_t_ is the mass of sample after treatment and W_0_ is the mass of sample before treatment.

#### 2.3.2. Scanning Electron Microscopy (SEM) Analysis

Small blocks of 5 (R-radial direction) × 5 (T-tangential direction) × 2 (L-longitudinal direction) mm in size were cut from the center of untreated and treated samples, and their cross sections were polished with a microtome. After ion-sputtering with gold, the gold coated portion was studied with SEM (FEI-XL30, Hillsboro, OR, USA) at 20 keV.

#### 2.3.3. Fourier Transform Infrared Spectroscopy (FTIR) Analysis

Chemical changes and bonding force between the silica and wood were analyzed using FTIR (Impact 410, Madison, WI, USA). Untreated and treated samples were performed with wave numbers ranging from 4000 to 400 cm^−1^ using 50 scans with a resolution of 4 cm^−1^.

#### 2.3.4. X-ray Diffraction (XRD) Analysis

The crystal structures of the untreated and treated samples were characterized using XRD (TTR3, Tokyo, Japan) patterns (Cu Kα radiation, λ = 0.154 nm) operating at 50 kV and 200 mA. The 2θ ranged from 5° to 60° with 0.02° scanning resolution. The crystallinity index (CrI, %) was determined as follows:

CrI = (I_200_ − I_am_)/I_200_ × 100%

where I_200_ is the adsorption peak intensity of the (200) reflection (at 2θ = 22.3°), and I_am_ is the minimum adsorption intensity between (110) and (200) peaks (at 2θ = 18°).

#### 2.3.5. Thermogravimetric (TG) Analysis

Shimadzu DTG-60 (Japan) was used for TG experiments to evaluate its thermal performance. About 5 mg of the sample was introduced into the sample pan and heated from room temperature to 600 °C with static air as a purge gas at a heating rate of 5 °C/min.

#### 2.3.6. Water Contact Angle Analysis

To evaluate the water-repellent or hydrophobicity properties of the composites, the tangential and radial contact angles (CAs) of the samples before and after treatment were measured with a CA-W type automatic contact angle meter. For the measurement of the static contact angle, a droplet volume of 5 μL was selected. The dynamic contact angle was continuously measured over 20 s.

#### 2.3.7. Mechanical Test

According to the three-point bending test method described in the Chinese national standard GB/T 1936.1-2009 (method of testing in bending strength of wood), the bending strength and elastic modulus of the wood before and after treatment were measured. The sample size was 300 (L) × 20 (T) × 20 (R) mm, and the load loading speed was 3 mm/min. The surface hardness change before and after the treatment of wood was measured in accordance with the Brinell hardness method in GB/T 1941–2009 (method of testing in hardness of wood). The sample size was 70 (L) × 70 (T) × 50 (R) mm. A steel ball with a diameter of 5.64 mm was pressed into the tangential section of the wood, and the loading rate was 5 mm/min until the indentation depth was 2.82 mm. The number of specimens for each test was selected according to the method in GB/T 1929–2009.

## 3. Results

### 3.1. Mass and Microstructure of the Developed Composites

**Figure 1:**
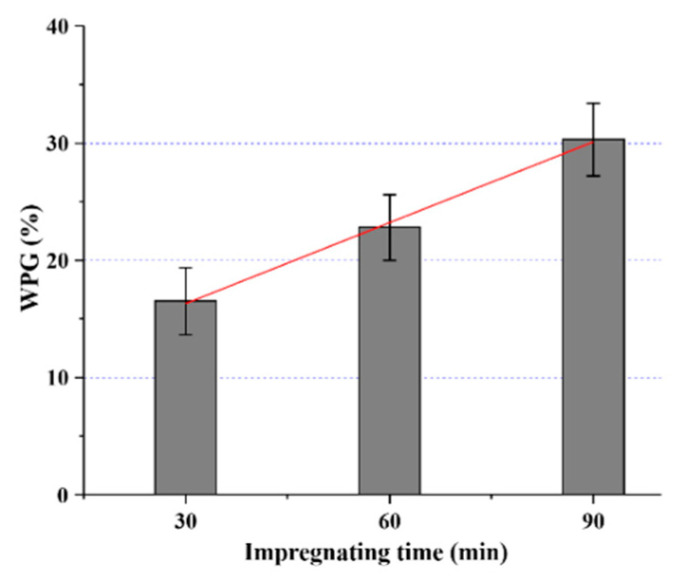
Weight percent gain (WPG) values of samples treated at different impregnating times under vacuum/pressure.

The average change in mass (averages of 24 replicates) of the developed SiO_2_-wood composites at different impregnating times is shown in Figure 1. It can be seen that the mass increased linearly with the increase of impregnating time (r^2^ = 0.99). The mean values of SiO_2_-wood composites mass increased from 16.5% to 30.3% (after 90 min impregnation) after curing, with a standard deviation of 3.1% and 6.6% and with a coefficient of variation of 0.188 and 0.218, respectively.

**Figure 2:**
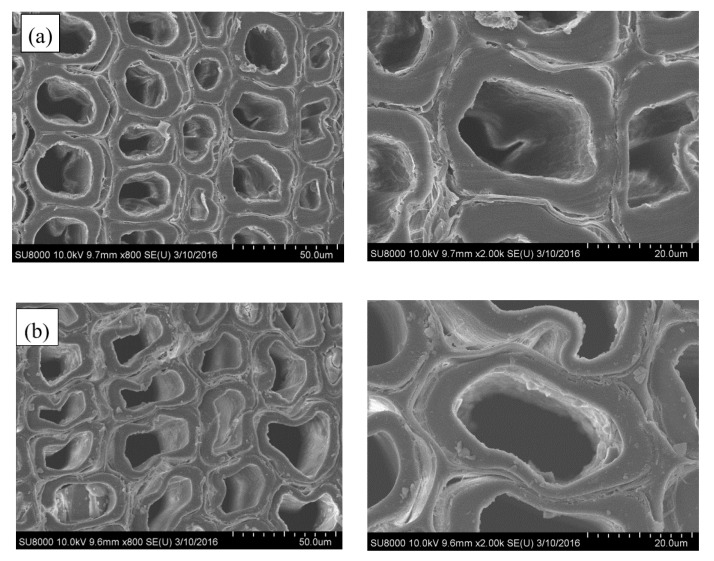

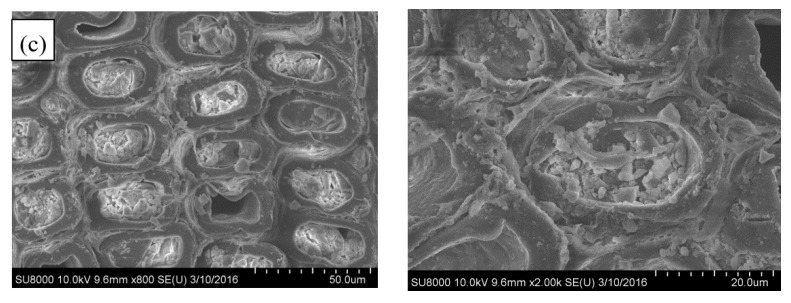
SEM micrographs of (**a**) untreated wood and the SiO_2_-wood composites with (**b**) 30 min and (**c**) 90 min impregnating time.

It is evident that the untreated sample is highly porous (Figure 2a). However, after a short time (30 min) of silica sol impregnation, the cell walls and a few tracheids were combined/filled with silica gel, and the internal surfaces of lumens were covered with an uneven layer of silica gel. Meanwhile, similar to the natural porous structure of untreated wood, the SiO_2_-wood composite with 30 min impregnation almost maintained cellular structure (Figure 2b). When the impregnating time was increased, the amount of SiO_2_ gel deposited on the cell wall increased, and the sample weight increased. The increase in weight is mainly caused by the increase in the content of silica filled in the cell lumens, and the content of silica adhered to the cell wall is less. The microstructures of SiO_2_-wood composite with 90 min impregnating is shown in Figure 2c; it is apparent that significant polycondensed SiO_2_ gels can be observed in the cell lumens. This may be due to the substantial Si–O–Si cross-linking framework formed.

### 3.2. Characteristics and Schematic of Chemical Bonding

**Figure 3:**
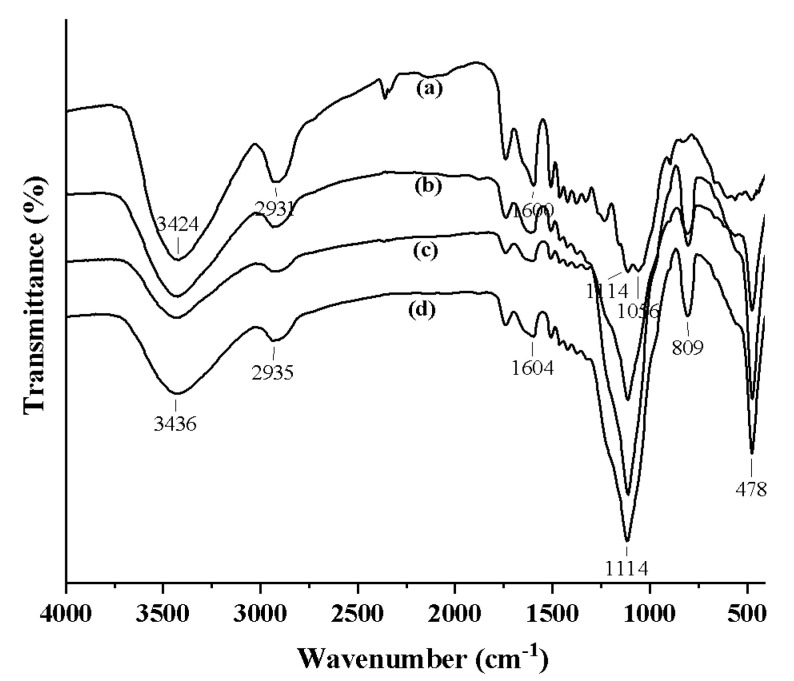
FTIR spectra of (**a**) untreated wood and the SiO_2_-wood composites with (**b**) 16.5%, (**c**) 22.8% and (**d**) 30.3% WPGs.

The FTIR spectra of the treated and untreated samples are shown in Figure 3. It is interesting that the band at 3421 cm^−1^ stretching vibration of –OH in the untreated wood is replaced by the stretching vibration band at 3432 cm^−1^ for SiO_2_-wood composite. The band intensity is also decreased. These observations indicate that the –OH reaction groups of wood may combine with silica sol to form Si–O–C covalent bonds. Bands at 2927 cm^−1^ (–C–H stretching) and 1596 cm^−1^ (–OH bending) were almost unchanged for wood and SiO_2_-wood composite. However, the Si–O–Si bands of SiO_2_-wood composite gave three characteristic bands (Figure 3b): a rocking vibration at 474 cm^−1^, a bending vibration at 806 cm^−1^, and an asymmetrical stretching vibration at 1110 cm^−1^. Due to the influence of broad and intensive Si–O–Si bonds of silica sol at 1110 cm^−1^, the –OH association band in cellulose and hemicellulose at 1110 cm^−1^ overlapped with the Si–O–C bonds, whereby the –OH groups of wood may have combined with silica sol to form the Si–O–Si and/or Si–O–C covalent bonds.

**Figure 4:**
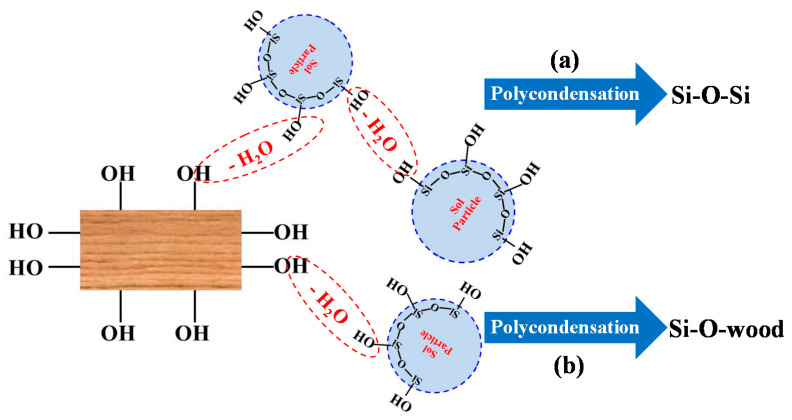
Schematic diagram of SiO_2_-wood composite formation, (**a**) reaction with –OH groups of silica sol, (**b**) reaction with the –OH groups of cell wall components.

Further to the FTIR results, the schematic of SiO_2_-wood composite formation is shown in Figure 4. Under external vacuum and pressure, the impregnating silica sol could flow through wood cells and coat the cell wall to some extent. Vacuum and pressure have important functions in these processes; the initial vacuum could firstly impregnate the wood cell wall quickly and uniformly. Pressure then increases the wood impregnation efficiency of silica sol and promotes the chemical reaction between the –OH groups of the silica sol and the –OH groups of cell wall components (Figure 4b) and also between the –OH groups of silica sol (Figure 4a). The main reaction sites are the –OH groups of the cell wall polymer, which includes cellulose, hemicellulose, and lignin. The chemical reaction could also take place in the lumen of the wood. It is worth noting that the aperture of the lumen is considerably larger than the size of the cell wall pores. The deposited silica sol is unstable in the cell lumens, and the final vacuum could eliminate the extra uncondensed silica sol in lumens. Further oven drying after the vacuum process for the final curing ensures the establishment of the Si-O-wood bonds and Si-O-Si cross-linking framework.

**Figure 5:**
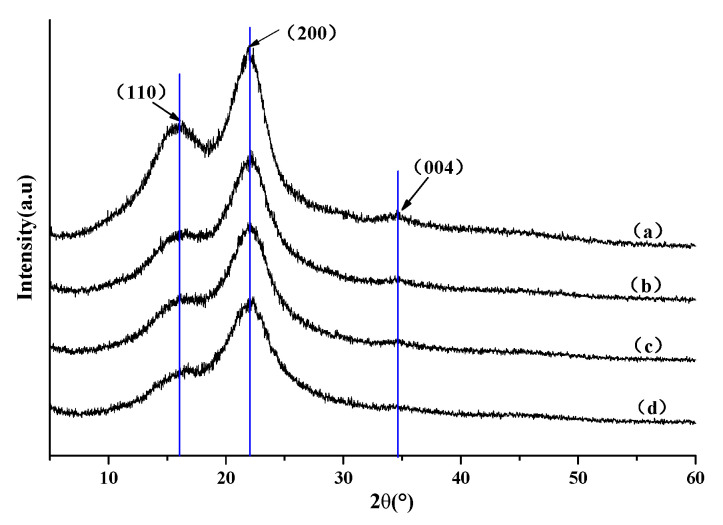
XRD patterns of (**a**) untreated wood and the SiO_2_-wood composite with (**b**) 16.5%, (**c**) 22.8% and (**d**) 30.3% WPGs.

The chemical bonding may result in internal restructure of the composites. The X-ray diffraction curves of untreated wood and SiO_2_-wood composites with different WPGs indicate that the 2θ diffraction curves of all the samples are similar, and the intensity is not much different (Figure 5). The XRD patterns for all materials show three peaks at 2θ values of 16.7°, 22.8°, and 34.5°, corresponding to the (110), (200), and (004) diffraction plane of cellulose Iβ pattern [34]. It can be seen that the nano-silica sol impregnation treatment did not destroy or even change the crystal structure of the wood samples. With the increase of WPG, the diffraction peak intensity and relative crystallinity decreased, which may be attributed to the presence of amorphous SiO_2_ in the cell wall of wood. The crystallinity of untreated wood and treated wood with 16.5%, 22.8%, and 30.3% weight gain rates were 45%, 40%, 37%, and 32%, respectively.

### 3.3. Thermal Properties of SiO_2_-Wood Composites

**Figure 6:**
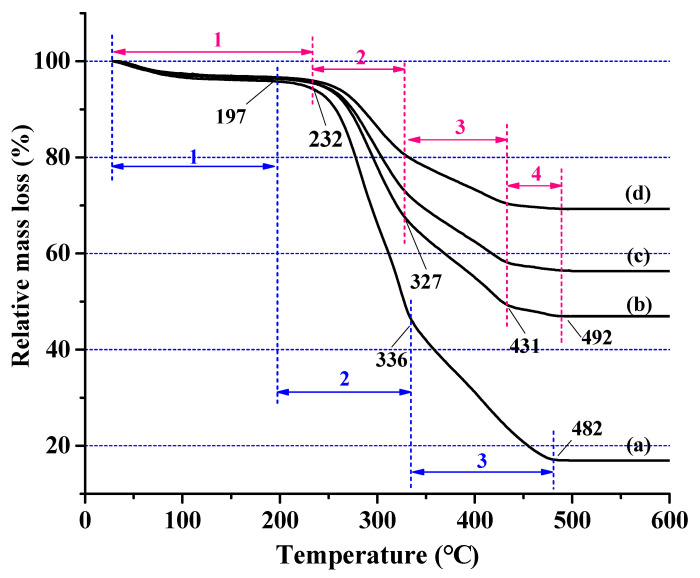
Thermogravimetric (TG) curves of (**a**) untreated wood containing (1) slight mass loss stage, (2) abrupt mass loss stage, (3) aromatization and carbonization stage (blue region) and the SiO_2_-wood composites with (**b**) 16.5%, (**c**) 22.8%, and (**d**) 30.3% WPGs, containing (1) slight mass loss stage, (2) abrupt mass loss stage, (3) remarkable mass loss stage, (4) slowly mass loss stage (red region).

TGA curves are used to determine the thermal stability of wood and wood materials. As the temperature increases, the materials begin to pyrolyze and the residue content increases, which indirectly means that the amount of volatile combustion products may decrease. Based on the mass loss observed in the TG curve, the untreated wood can clearly be divided into three different temperature regions (Figure 6): from room temperature to 197 °C, only a small amount of mass loss occurs in the material (region 1), and the mass loss was about 4.2%, which was mainly due to the evaporation of absorbed moisture and the partial decomposition of hemicellulose. An abrupt mass loss (region 2) occurred at 197–336 °C, which was mainly caused by hemicellulose decomposition, accompanied the continuous decomposition of cellulose and lignin, and mass loss reached 55.0%. Starting from 336 °C, all components of the wood gradually degraded leading to aromatization and carbonization (region 3). After 482 °C, the wood was completely carbonized, and 17.1% carbon residue was left.

Compared with the untreated wood, the SiO_2_-wood composites had distinctive TG curves, characteristically, of four different temperature regions: in region 1, a slight mass loss was observed from room temperature to 232 °C, and the mass loss was almost the same as that of untreated wood. The mass loss in region 2 decreased rapidly from 232 °C to 327 °C, followed by another remarkable mass loss (region 3) at 327–431 °C. The most interesting feature about the TG curves of the SiO_2_-wood composites was the temperature from 431 °C to 492 °C (region 4), compared with untreated wood, in which the mass loss declined slowly. The ultimate decomposition temperature of SiO_2_-wood composites shifted towards the higher temperature, after which lower rates of weight loss were observed. Eventually, higher amounts of residues of 47.0%, 56.5%, and 69.3% were obtained for 16.5, 22.8 and 30.3 WPG, respectively. The residue produced by SiO_2_ wood composites depends on the WPG of the composites, and the residual amount increases with the increase of WPG. This result shows that the silica sol impregnated in wood can withstand higher temperatures and can improve the thermal properties of wood.

### 3.4. Hydrophobicity of Composites

**Figure 7:**
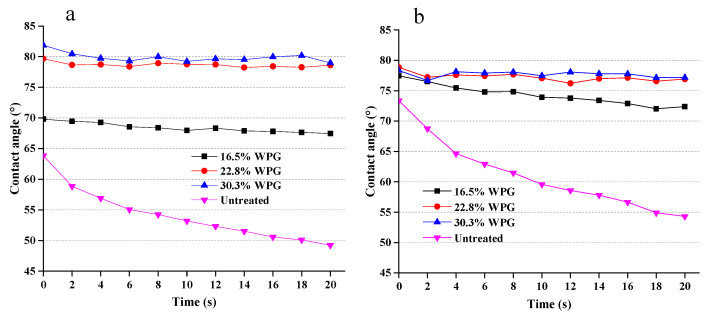
Water contact angles of SiO_2_-wood composites on tangential (**a**) and radial (**b**) sections.

Figure 7 shows the water contact angles of tangential and radial sections of untreated wood and composites of different WPG over a time period of 20 s. It is evident that the instantaneous contact angles of tangential sections of composites increased with increasing WPG (Figure 7a). The instant contact angle increased by about 10% for the composite with 17% WPG, 25% for that with 23% WPG and 30% for that with 30% WPG. It is very interesting that while the contact angle of untreated wood consistently reduced by the duration of wetting, from 63.8° to 49.2°, the contact angles of composites is almost independent of the wetting time. Therefore, at the end of 20 min wetting, the composites with 23% WPG increased the contact angles by 61% compared to the matched wood materials.

The instantaneous contact angle of the composite radial section is slightly higher than that of the untreated wood (Figure 7b). It is also interesting that the loading of WPG had little effect on the instantaneous contact angles. However, after 20 s of wetting, the water contact angle of untreated wood decreased from 73.4° to 54.3°. By contrast, the contact angles of composites almost remained unchanged throughout the exposure of water wetting. Therefore, at the end of 20 min, the contact angle of the composite with 30.3% WPG increased by 42% compared to the matched wood. These results indicate that silica sol impregnated composites can improve the water repellency of the materials and have good hydrophobicity.

### 3.5. Mechanical Properties of the Composites

**Figure 8:**
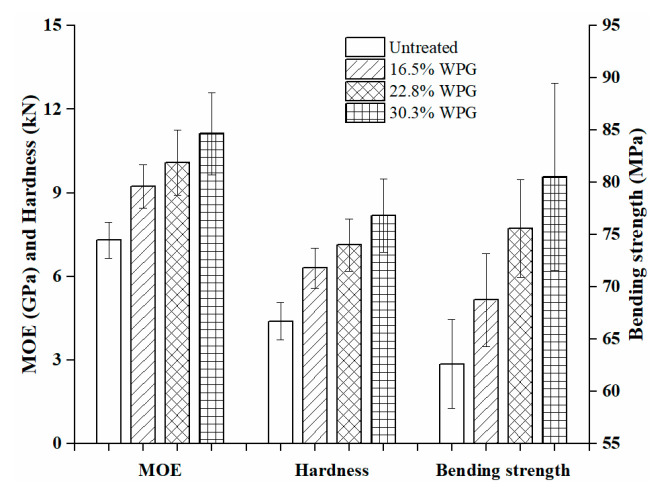
The mechanical properties of the untreated wood and the SiO_2_-wood composites.

In order to evaluate the reinforcing effect of nano silica sol, mechanical properties were measured. The modulus of elasticity, bending strength and hardness of composites and untreated wood are the average of 62, 35, and 62 replicates, respectively, which are summarized in Figure 8. The mean values for modulus of elasticity of the composites at three WPGs of 16.5%, 22.8%, and 30.3% were 9.23, 10.08, and 11.12 GPa, with a standard deviation of 0.78, 1.16, and 1.47 GPa and with a coefficient of variation of 0.085, 0.115, and 0.132, respectively, while the modulus of elasticity of the untreated wood was 7.30 (±0.64) GPa. This means that the stiffness of the 30.3% WPG composite is increased by 52.3%. The bending strength of the composite with 30.3% WPG was 80.51 (±8.96) MPa, which is 28.6% higher than the untreated 62.62 (±4.24) MPa. The hardness of composite materials increases with the increase of WPG. When WPGs were 16.5%, 22.8%, and 30.3%, the average hardness of composites was 4.20, 4.75, and 5.46 kN, with a standard deviation of 0.48, 0.62, and 0.88 kN and with a coefficient of variation of 0.114, 0.131, and 0.161, respectively, which were increased by 43.7%, 62.1%, and 86.3%, respectively, compared with the average hardness of untreated wood. The mechanical properties of composites are linearly related to WPG. The improvement of the mechanical properties is mainly due to the impregnation of the silica sol into the wood to strengthen the wood cell walls or fill the voids of the wood structure and their combined effects between wood and silica sol through various physical, chemical, and mechanical interactions/bonding.

## 4. Conclusions

In-situ SiO_2_-wood composites have successfully been developed by introducing commercial nano silica sol into wood. The in-situ combination of wood and silica sol was efficient and effective with the aid of vacuum impregnation, depending on the duration, with the mass and the amounts of SiO_2_ gel of the composites increasing with the increasing of impregnation time. The composites almost maintained cellular structure, and the SiO_2_ gel was significantly combined and extended from the surface of the cell lumens, while a small portion of silica sol penetrated into and developed bonds with the cell walls. The processing technologies stimulated the –OH groups of wood, which combined with silica sol to form the Si–O–Si and Si–O–C covalent bonds, resulting in the transformation of the microstructure of wood to composites and hence significant improvements in the thermal and mechanical properties of composites. The hydrophobicity of the developed composites is much lower than that of raw wood materials.
